# Effects of human disturbance on postnatal growth and baseline corticosterone in a long-lived bird

**DOI:** 10.1093/conphys/coab052

**Published:** 2021-07-08

**Authors:** Hannah Watson, Pat Monaghan, Britt J Heidinger, Mark Bolton

**Affiliations:** 1 Institute of Biodiversity, Animal Health and Comparative Medicine, University of Glasgow, Glasgow, G12 8QQ, UK; 2 Evolutionary Ecology, Lund University, SE-223 62 Lund, Sweden; 3 Biological Sciences Department, North Dakota State University, Fargo, ND 58108, USA; 4 RSPB Centre for Conservation Science, The Lodge, Sandy, Bedfordshire, SG19 2DL, UK

**Keywords:** Catch-up growth, conservation physiology, CORT, developmental plasticity, glucocorticoids, human disturbance, non-linear growth, tourism

## Abstract

Prolonged or repeated episodes of environmental stress could be especially detrimental for developing young, via impaired growth or development. Despite this, most studies investigating the effects of human recreational and tourism activities have focused on adults. An increasing demand for nature-based tourism in remote locations means that many seabirds, which have evolved largely in the absence of predators and humans, are being exposed to novel pressures. The slow-growing semi-precocial nestlings of the European storm petrel *Hydrobates pelagicus* experience higher mortality rates in nests exposed to human recreational disturbance. Here, we examine whether surviving nestlings reared in disturbed areas are also affected via changes in growth trajectories and baseline circulating glucocorticoids. Nestlings reared in high-disturbance areas displayed delayed mass growth, and we found weak evidence for slower rates of mass gain and tarsus growth, compared with nestlings reared in undisturbed areas. There were no differences in wing growth, consistent with prioritization of long wings, important for post-fledging survival. A tendency for a less marked age-related decline in corticosterone (CORT) in disturbed nestlings offers limited evidence that changes in growth trajectories were mediated by baseline CORT. However, disturbed nestlings could have experienced overall higher GC exposure if the acute GC response was elevated. ‘Catch-up’ growth enabled high-disturbance nestlings to overcome early constraints and achieve a similar, or even larger, asymptotic body size and mass as low-disturbance nestlings. While catch-up growth has been shown to carry costs for parents and offspring, the effects of disturbance were slight and considerably smaller than growth alterations driven by variation in environmental conditions between years. Nonetheless, effects of human recreational activities could be exacerbated under higher levels of human disturbance or in the presence of multiple pressures, as imposed by present rapid rates of environmental change.

## Teaser

The effects of human disturbance on avian physiology have been mainly studied in adults, yet consequences for developing young could be severe. Despite early reduction in growth rates, storm petrel nestlings in disturbed areas fledged at the same size as undisturbed chicks and showed no marked elevation in baseline glucocorticoids.

## Introduction

Free-living organisms are currently being exposed to unprecedented rates of anthropogenic-induced environmental change, transforming and disrupting terrestrial and marine ecosystems (Halpern *et al.*, 2019; Nolan *et al.*, 2018). It is well understood that human activities, which are linked to urbanization and agriculture, can have marked effects on a suite of phenotypic traits and survival of wild animals (Alberti *et al.*, 2017). Although generally considered to be less intrusive, it has been shown that even non-consumptive wildlife activities, such as wildlife-watching, can negatively impact organisms via changes to behaviour (Beale and Monaghan, 2004), physiology (Romero and Wikelski, 2002), reproductive success (Watson *et al.*, 2014) and energy expenditure (Williams *et al.*, 2006). However, effects of human disturbance associated with recreation and tourism are often context dependent, varying with age, sex, life-history stage or experience (Creel *et al.*, 2002; Dantzer *et al.*, 2014; Spercoski *et al.*, 2012; Walker *et al.*, 2005, 2006) of the affected animals and the nature and intensity of the disturbance (French *et al.*, 2017). In some cases, ecotourism can even exert positive effects, such as by reducing predation pressure (Larm *et al.*, 2020) and providing funding for conservation efforts (Kirkby *et al.*, 2011). As the demand for nature-based recreation and tourism increases worldwide (Flather and Cordell, 1995), there is an increasing need to understand its potential impacts for health and persistence of wild populations, especially in light of context dependency.

While organisms are well adapted to cope with predictable changes in their environment, such as the transition from day to night or the changing of the seasons (Foster and Kreitzman, 2005), unpredictable perturbations present a greater challenge requiring a sophisticated suite of responses. In vertebrates, the response to an unpredictable challenge is mediated by the hypothalamic–pituitary–adrenal (HPA) axis and its regulation of levels of glucocorticoid (GC) hormones. The HPA axis is highly conserved across vertebrates, highlighting the evolutionary importance of optimal management of GC levels (Romero, 2004). Unpredictable stressors stimulate a short-term increase in secretion of GCs, which subsequently prioritizes activities that facilitate survival, while suspending or down-grading ‘non-essential’ activities (Wingfield and Kitaysky, 2002). Although the GC stress response is clearly adaptive, if the stress exposure is prolonged or repeated, sustained diversion of energy away from growth and maintenance processes, such as immune function, can be detrimental (Blas and Bortolotti, 2007; Davison *et al.*, 1983; Kitaysky *et al.*, 2003; Sapolsky, 2015; Sapolsky *et al.*, 2000). The consequences of long-term or repeated stress exposure could thus be particularly severe in developing young, directing resources away from essential processes, such as growth and healthy organ development. Indeed, it is well understood that exposure to stressful environmental conditions and excess GCs during early life can give rise to fundamental and lifelong effects on an individual’s phenotype, leading to increased disease susceptibility and a shorter lifespan (Harris and Seckl, 2011; Monaghan and Haussmann, 2015). Furthermore, since body mass at independence is a strong predictor of survival in natural environments (e.g. Krementz *et al.,* 1989; McMahon *et al.*, 2000), constraints on developmental growth rates imposed by excess GC exposure could result in cascading effects on recruitment, with negative consequences at the population level.

Despite the potential for stress exposure during early life to lead to lifelong detrimental effects, to date, most studies of the effects of human recreational and tourism activities on animals have focused on the effects on adults (e.g. Creel *et al.*, 2002; French *et al.*, 2010; Spercoski *et al.*, 2012; Strasser and Heath, 2013). Chronic human disturbance could affect developing young via direct exposure to disturbance or indirectly via changes in parental behaviour. Effects of human activities on young could subsequently be manifest as changes in levels of circulating GCs and/or growth rates. However, studies have failed to reveal consistent effects of human disturbance on offspring physiology and growth. While some studies reveal higher baseline GCs and a stronger GC stress response (Almasi *et al.*, 2013; Angelier *et al.*, 2016), others find neither baseline or stress-induced GCs to be affected by human activities (Strasser and Heath, 2011), while another study reported no differences in baseline GCs but stronger acute GC responses (Walker *et al.*, 2005). The lack of a uniform pattern in how chronic stress, such as human disturbance, affects GC levels, could be explained by large variation between individuals (Cockrem and Silverin, 2002) and species (Dantzer *et al.*, 2014) as well as the context, e.g. type and/or level of disturbance (Almasi *et al.*, 2013; French *et al.*, 2017). Similarly, while some studies have demonstrated reduced body mass of young reared in areas of high human disturbance (Albores-Barajas *et al.*, 2009; Almasi *et al.*, 2013; McClung *et al.*, 2004; Müllner *et al.*, 2004; Remacha *et al.*, 2016), other studies have found no direct effects of human disturbance on body mass at independence (Engelhard *et al.*, 2002; Yorio and Boersma, 1992). Nonetheless, there could be hidden effects on growth trajectories that are not revealed by measuring body mass only at independence, if, for example, compensatory growth strategies are incurred enabling disturbed young to overcome early slow growth and achieve the same body size at independence as undisturbed individuals.

Many seabirds breed in remote or inaccesible locations and thus have evolved in the absence of predators and humans. While this may have once afforded them greater protection from the impacts of expanding human activities, an increasing demand for tourism in more remote environments (Dawson *et al.*, 2011), combined with the loss of wilderness areas at more than twice the rate of protection (Watson *et al.*, 2016), presents a growing concern for potential impacts of ecotourism on wildlife in remote locations. Despite nesting in cavities, out of sight, our previous research revealed that daily human recreational disturbance is associated with increased nestling mortality (presumably due to starvation and parental desertion) in the European storm petrel *Hydrobates pelagicus* (hereafter, storm petrel) (Watson *et al.*, 2014). Both adults and nestling storm petrels are vulnerable to disturbance from daytime human activities during the long periods of incubation and chick rearing, respectively, and effects of disturbance on young could be enacted directly or indirectly, via effects on parental condition and/or parental care.

In the present study, we examined whether surviving storm petrel young reared in areas exposed to tourism activities are also affected via changes in baseline levels of circulating GCs and/or growth trajectories, compared with nestlings reared in areas subject to no visitor pressure. We previously found that, although unfavourable environmental conditions in nestlings were associated with short telomeres and accelerated attrition rates (both markers of stress exposure and survival probability), telomere dynamics were independent of human disturbance (Watson *et al.*, 2015). Since telomere loss is accelerated by elevated GC exposure (Monaghan, 2014), we therefore predict that circulating GCs vary between years in response to varying environmental conditions but not in relation to human disturbance.

## Materials and methods

### Study system

The study was conducted at the island of Mousa, located in the Shetland archipelago, UK (60°0’N, 1°10’W), which supports >11 000 pairs of storm petrels breeding in natural cavities. Mousa receives 4000–5000 visitors during the breeding season due to its wildlife and archaeological interests. Visitor activity (on foot) is concentrated temporally, during daylight hours, and spatially, within a 3.2-km circular footpath in the centre of the island. We considered nests located within 10 m of the footpath to be subject to ‘high’ levels of visitor pressure and nests >150 m from the footpath to be subject to ‘low’ levels of visitor pressure. Study nests were equally distributed among six replicated plots, three in each of the high- and low-disturbance categories. Nests were located in either stone walls (linear features up to 200 m) or rock scree (non-linear features up to 450 m^2^), and both of these nesting habitat types were equally represented within each of the high- and low-disturbance plots. There is some visitor activity at night, specifically focussed on viewing storm petrels, though this is tightly regulated with infrequent guided tours operating within the existing footprint of diurnal human activity.

Storm petrels lay a single egg and share incubation, brooding and provisioning duties. While they are strictly nocturnally active at the colony—only leaving or returning to the nest cavity during darkness—they remain in the nest for several days at a time during incubation and brooding, and thus both females and males are vulnerable to disturbance from daytime human activities. Storm petrel nestlings are semi-precocial: they hatch with a thick layer of down and become thermally independent at an early age, but they do not leave the nest until fledging, and prior to this are completely dependent on the parents for food. Once brooding has ceased, the single semi-precocial nestling remains alone within the confines of the nest cavity during the day, while the adults forage far out at sea, returning to the colony to provision the chick at night. Although storm petrels remain out of visual contact with humans, they are exposed to noise, vibrations and odours associated with tourist activity close to or directly above their nests. Importantly, both incubating/brooding adults and chicks are unable to flee and thus avoid a disturbance event. Due to their slow life histories (incubation c. 40 d, chick rearing c. 65–70 d), adults and young can be exposed to repeated visitor disturbance at the underground nest for more than 6 and 9 wk, respectively. By studying a species that produces only one offspring per breeding attempt, we were able to eliminate the potentially confounding factor of sibling competition on growth and physiological development (Love *et al.*, 2003).

### Data collection

Biometrics were recorded regularly from 55 nestlings that survived through to fledging in 2010 and 2011. Mass, tarsus length and wing length (once the outermost primary feather emerged from the quill sheath at c. 30 d) were measured approximately every 6 d between the ages of 7.51 ± 0.72 and 50.6 ± 0.95 d (means ± SE; *n* = 55 nestlings), yielding 10.5 ± 0.69 measurements per individual. To account for differences in timings of weighings, mass was corrected to a standardized time (18:00) according to the age-related rate of proportional weight loss (see Bolton 1995). Blood (70 μl) was collected by venepuncture of the brachial vein at two points during development, with repeated samples obtained from 41 nestlings, and a median of 31 d elapsed between repeated measures (range: 22–46 d). All blood samples were collected within 3 min of the onset of disturbance (determined as when the investigator approached to within 1 m of nest), which is considered representative of baseline corticosterone (CORT, the primary GC in birds) (Romero, 2004); there was no effect of blood-sampling time on CORT levels (*t* = 1.24, *P* = 0.218). Blood samples were separated by centrifugation; plasma and red blood were stored at <5°C in the field for a maximum of 3 d before being transferred to −20°C. Procedures were carried out under licence from the UK Home Office and Scottish Natural Heritage. It was considered too disturbing to perform a standardized capture-restraint protocol, followed by a second blood sample for the measurement of acute CORT levels.

Plasma CORT was determined by enzyme immunoassay (Enzo Life Sciences Inc.). CORT was first extracted from plasma with 2 ml of diethyl ether in a dry-ice/methanol bath, evaporated under nitrogen gas and re-suspended in 300 μl of the assay buffer. Prior to extraction, plasma was spiked with 20 μl 150 cpm [^3^H]-CORT to calculate recovery rate. Average recovery of samples was 84.5%. Samples were run in duplicate at a 1:20 dilution, and repeated samples were run on the same plate. A standard curve was included on each plate with six standards (in triplicate) ranging from 20 000 to 15.63 pg ml^−1^. After adding stop solution, absorbance was measured immediately using a Thermo Multiskan EX plate photometer at 405 nm and corrected for 570 nm. Values were corrected for initial plasma volume and individual recovery. Mean inter-assay and intra-assay coefficients of variation were 14.5% and 12.8%, respectively. The detection limit of the assay was 0.46 ng ml^−1^; this was calculated by taking two standard deviations away from the mean of the total-binding wells. One sample fell below the detection limit and was assigned the detection limit. DNA was extracted using the NucleoSpin Blood kit (Macherey-Nagel, Germany) for molecular sex determination (Griffiths *et al.*, 1998).

### Statistical analysis

All statistical analyses were performed in R 4.0.0. While wing growth was linear (see below), growth in respect of body mass and tarsus did not follow a linear pattern during the period of observations, and therefore non-linear mixed-effects models were fitted to data on body mass and tarsus length using the package FlexParamCurve (Oswald *et al.*, 2012). This allowed us to test for variation in nestling growth trajectories in relation to human disturbance. A three-parameter Gompertz function describing the relationship between mass (g) or tarsus length (mm) and age (d) as a function of *A* the asymptotic mass or tarsus length, *k* the growth rate constant and *i* the inflection point (age at maximum growth rate) was fitted to data. The optimal random effects structure was identified by allowing different combinations of the three curve parameters to vary randomly with nestling identity, while the fixed component remained saturated; models were fitted by restricted maximum likelihood and compared using the Akaike Information Criterion (AIC). The optimal random effects structure allowed the asymptote and inflection point to vary randomly with nestling identity. Saturated models included the fixed effects of disturbance (high or low), sex (female or male), hatching date (a covariate to test for within-season variation) and year (a two-level fixed factor accounting for inter-annual variation). Seasonal and inter-annual variations in the effect of disturbance on dependent variables were considered by including the respective interactions with hatching date and year. In non-linear growth models, all fixed effects were allowed to affect all three parameters of the curve, except hatching date and its interactions; since there were fewer observations in late development for later-hatched nestlings, hatching date was not allowed to affect the asymptote *A*. The shape parameter *m* was fixed at the mean across the dataset: 2.88 for tarsus and 1.065 for mass.

Linear mixed models (LMMs) with a normal error structure and a random effect of nestling identity were fitted to data on wing length and log-transformed nestling CORT using the package lme4. LMMs for wing length and CORT included the fixed effects of age, disturbance (high or low), sex (female or male), hatching date (a covariate to test for seasonal variation) and year (a two-level fixed factor accounting for inter-annual variation). To check whether the effect of disturbance varied with age, the two-way interaction between age and disturbance was included. Seasonal and inter-annual variations in the effect of disturbance on dependent variables were considered by including the respective two-way interactions with hatching date and year. The saturated model for CORT also included body mass and time of day.

For both non-linear and linear models, the optimal fixed structure was obtained by stepwise deletion comparing models fitted by maximum likelihood, using maximum likelihood ratio tests and eliminating terms when *P* > 0.1. All results are presented as means ± SE (unless otherwise stated) from minimum-adequate models fitted by restricted maximum likelihood. Model fits were assessed by visualization of residual plots. The significance of parameter estimates was generated based on the *t*-distribution. Interaction contrasts were assessed using the emmeans package and adjusted for multiple comparisons using the false detection rate. Confidence intervals (95% CIs) for parameter estimates from LMMs were calculated using the effects package. Since reliable confidence intervals for non-linear data cannot be derived in the same way as for linear data, uncertainty around parameter estimates for fixed effects from non-LMMs was assessed by bootstrapping with 1000 iterations using boot_nlme in the nlraa package.

## Results

### Growth and human disturbance

**Figure 1:**
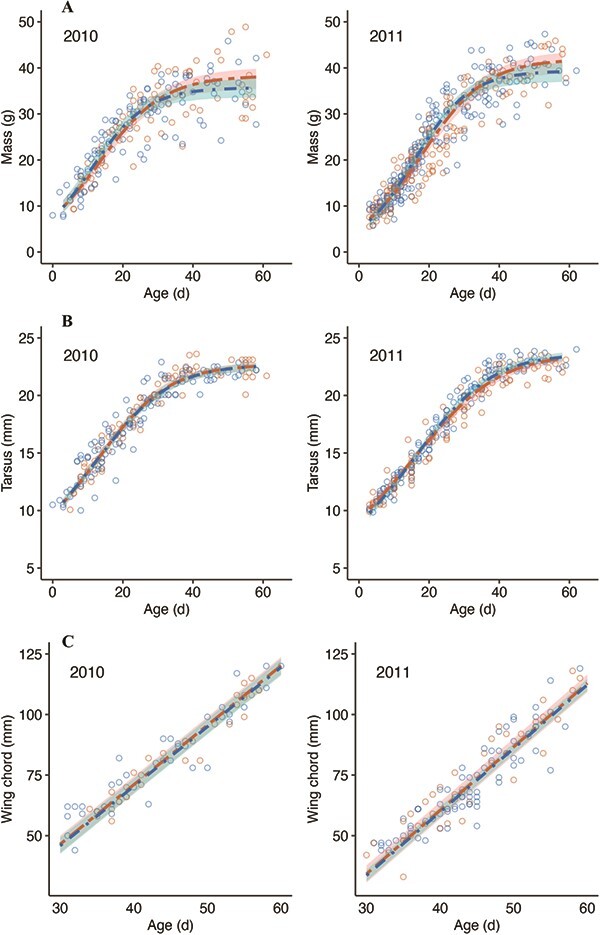
Growth trajectories for **(A)** mass, **(B)** tarsus and **(C)** wing of storm petrel nestlings (*n* = 55) reared in nests subject to high (orange; ≤10 m from footpath) and low (blue; >150 m from footpath) levels of human tourist disturbance in two consecutive years of study. Bold dashed lines represent predictions for the average hatching date, derived from minimum-adequate mixed models fitted within the range of observed values; shading represents 95% CIs; and circles indicate observed values. CIs for non-linear models were generated by bootstrapping model predictions (*n* = 1000). Despite best-fit models suggesting that nestlings reared in the presence of high human disturbance exhibited lower growth rates of mass and tarsus (2011 only), a later inflection point of mass growth and higher asymptotic mass, the only parameter where 95% CIs of bootstrapped estimates did not overlap with zero was the inflection point for mass.

All three parameters describing the rate of mass gain in nestlings were significantly affected by human disturbance (Fig. 1A): nestlings reared in low-disturbance nests exhibited an earlier inflection point (*ß* = −2.17 ± 0.76, *t*_513_ = −2.85, *P* = 0.005), higher growth rate constant (*ß* = 1.78^e-2^ ± 7.02^e-3^, *t*_513_ = 2.53, *P* = 0.01) and lower asymptotic mass (*ß* = −2.57 ± 1.22, *t*_513_ = −2.10, *P* = 0.04), compared with those reared in the presence of high human disturbance. However, bootstrapping of parameter estimates revealed that the 95% CIs around the parameters *A* (95% CIs: −4.92 to 0.48) and *k* (95% CIs: −1.40^e-3^ to 3.69^e-2^) for low disturbance nestlings overlapped with zero, while CIs around the parameter *i* did not overlap with zero (95% CIs: −3.73 to −0.28). The effects of disturbance on mass growth were consistent between years (all *P* > 0.3). The only parameter of tarsus growth affected by human disturbance was the growth rate constant (*k*), and the effect differed between the 2 years of the study (Fig. 1B; disturbance _low_:year_2011_: *ß* = 1.12^e-2^ ± 5.65^e-3^, *t*_388_ = 1.99, *P* = 0.05). The growth rate constant of nestlings reared in low disturbance sites did not differ from high-disturbance nestlings in 2010 (contrast: −2.45^e-3^, *P* = 0.6) but was significantly higher in 2011 (contrast: 8.78^e-3^, *P* = 0.008). However, again, 95% CIs of bootstrapped estimates of *k* ~ disturbance_low_:year_2011_ overlapped with zero (−1.60^e-3^ to 2.62^e-2^). Neither the asymptote (*t*_387_ = 1.03, *P* = 0.3) nor inflection point (*t*_384_ = −0.67, *P* = 0.5) of tarsus growth varied between disturbance levels. The average nestling (averaged across hatching date and years) from a high-disturbance nest exhibited *k* values that were 0.86 (mass) and 0.97 (tarsus) of an average nestling from a low-disturbance nest. On average, high-disturbance nestlings reached the inflection point 2.2 d later and took an extra 5 d to reach 90% of asymptotic mass, compared with low-disturbance nestlings. Neither absolute wing length (disturbance_low_: *ß* = −0.63 ± 1.83, *t*_74.0_ = −0.35, *P* = 0.7) nor the age-related change in wing length (age:disturbance_low_: *ß* = −1.99^e-2^ ± 5.40^e-2^, *t*_103.6_ = −0.37, *P* = 0.7) differed between nestlings reared in low- or high-disturbance sites (Fig. 1C).

### Growth and inter- and intra-annual variation

Growth trajectories for mass and tarsus significantly differed between the 2 years of study (Fig. 1A and B). In 2011, growth curves exhibited a later inflection (mass: *ß* = 4.75 ± 0.74, *t*_513_ = 6.43, *P* < 0.001; tarsus: 4.15 ± 0.57, *t*_388_ = 7.34, *P* < 0.001) and lower growth rate constant of tarsus (high disturbance only; contrasts: high-2011 vs high-2010 = −1.31^e-2^, *P* = 0.008; low-2011 vs low-2010 = −1.88^e-3^, *P* = 0.6) but reached a higher asymptote (mass: *ß* = 3.65 ± 1.11, *t*_513_ = 3.28, *P* = 0.001; tarsus: *ß* = 0.93 ± 0.26, *t*_388_ = 3.57, *P* < 0.001). Inter-annual variation was larger than variation due to human disturbance, and 95% CIs of bootstrapped estimates did not overlap with zero. Later-hatched nestlings in both low- and high-disturbance areas exhibited a lower growth rate constant of mass (*ß* = −1.04^e-3^ ± 3.57^e-4^, *t*_513_ = −2.92, *P* = 0.004) and a delay in the inflection point of both mass (*ß* = 6.96^e-2^ ± 3.24^e-2^, *t*_513_ = 2.15, *P* = 0.03) and tarsus (*ß* = 5.28^e-2^ ± 2.63^e-2^, *t*_388_ = 2.00, *P* = 0.05) growth curves, although bootstrapped 95% CIs all overlapped with zero. The rate of wing growth was significantly higher in 2011, compared with 2010 (Fig. 1C; age:year_2011_: *ß* = 0.17 ± 5.45^e-2^, *t*_104.2_ = 3.038, *P* = 0.003).

Sex did not affect any of the growth parameters relating to mass or tarsus (all *P* > 0.4). Neither wing length nor age-related change in wing length varied with sex (*P* = 0.6) or hatching date (*P* = 0.4). For mass and tarsus, respectively, the estimated variances (mean ± SD) associated with the random effect of nestling were 12.18 ± 3.49 and 0.53 ± 0.73 (*A*) and 3.25 ± 1.80 and 2.54 ± 1.59 (*i*), with a correlation of 0.57 and 0.025. For wing, the variance associated with the random effect of individual was 50.7 ± 7.12.

### Baseline CORT and human disturbance

**Figure 2:**
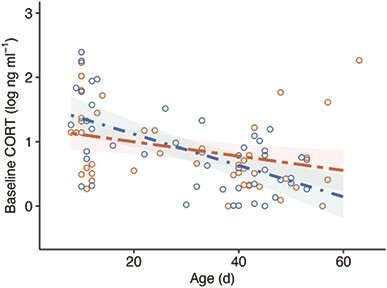
Baseline plasma CORT in storm petrel nestlings (*n* = 51) reared in nests subject to high (orange; ≤ 10 m from footpath) and low (blue; > 150 m from footpath) levels of human tourist disturbance. Baseline CORT declined with increasing age but the age-related change in CORT did not differ significantly between high and low disturbance nestlings (*P* = 0.06). Predicted values (bold dashed lines) from the model including age:disturbance are shown with 95% CIs (shaded bands). Circles indicate observed values.

While there was a tendency for a difference in the age-related change in baseline CORT between disturbed and undisturbed nestlings, the slopes were not significantly different from one another (Fig. 2; age:disturbance_low_: *ß* = −1.33^e-2^ ± 6.94^e-3^, *t*_59.1_ = −1.91, *P* = 0.06). Baseline plasma CORT decreased with increasing age during postnatal development in both low (mean slope: −2.44^e-2^, 95% CIs: −3.48^e-2^ to −1.40^e-2^) and high (mean slope: −1.11^e-2^, 95% CIs: −2.06^e-2^ to −1.73^e-3^) disturbance nestlings, with overlapping 95% CIs. The effect of disturbance on baseline CORT did not vary within (hatching date:disturbance_low_: *P* = 0.6) or between years (year_2011_:disturbance_low_: *P* = 0.6). There was no significant variation in baseline CORT associated with sex, hatching date, year, body mass or time of day (all *P* > 0.1). The variance associated with the random effect of individual was 1.36^e-2^ ± 0.12.

## Discussion

While environmental constraints on developmental growth have been widely documented (Dmitriew, 2011), there has been little consideration for how human activities associated with recreation and tourism might shape growth trajectories and subsequently affect fitness. Our results provide novel evidence for variation in postnatal development in birds driven by human disturbance associated with wildlife tourism and manifested as marginally delayed and slower growth followed by compensation prior to fledging. Nestlings reared in the presence of tourist activities were able to ‘catch up’ with those young that were not subject to disturbance, enabling them to fledge at a similar size, the benefits of which are likely to outweigh any long-term costs (e.g. reduced reproductive output or lifespan) associated with catch-up growth strategies (Metcalfe and Monaghan, 2001, 2003). It is important to emphasize that, in the present study, observed effects of disturbance on growth were small and associated with high uncertainty. Furthermore, there were no differences in wing growth between disturbed and undisturbed nestlings, consistent with the theory that wing growth is prioritized over mass due to its importance in post-fledging survival (Jones and Ward, 2020).

Despite small alterations in growth trajectories, there was limited evidence that human disturbance induced physiological stress in chicks. The observed age-related decline in baseline CORT was less marked in high-disturbance nestlings, though not significantly different to the decline observed in undisturbed nestlings. While repeated exposure to stress has often been associated with an elevation in baseline GCs, there is no consensus GC response to chronic stress (Dickens and Romero, 2013). Thus, we cannot rule out the possibility that nestlings reared in the presence of human disturbance became more stress reactive, since we did not measure acute levels of CORT. However, the previously reported absence of effects of human disturbance on telomere dynamics in nestling storm petrels (Watson *et al.*, 2015) could indicate that human disturbance does not increase overall GC exposure, which would otherwise be expected to accelerate telomere attrition rates (Herborn *et al.*, 2014). Consistent with this possibility, adult storm petrels nesting at the edges of the Italian island of Marettimo exhibited no differences in either baseline or stress-induced CORT, in association with exposure to traffic from tourist boats, compared with birds nesting at the island’s interior (Soldatini *et al.*, 2015). However, the response to stress can be multifaceted, and other physiological changes may occur.

While it is not certain if nestling storm petrels can elicit an acute adrenocortical response to a stressor, the young of related species have been shown to exhibit a robust acute stress response shortly after hatching, albeit greatly reduced compared with adults (Adams *et al.*, 2008; Fiske *et al.*, 2013; Quillfeldt *et al.*, 2009). Such hyposuppression of the HPA axis has been demonstrated in many vertebrates and is assumed to be adaptive, by preventing exposure to excess GCs during the vulnerable period of development (Wada *et al.*, 2007). In contrast to the aforementioned studies in related species, one study found that adult storm petrels did not elicit a stress response following a standardized capture-restraint protocol (Soldatini *et al.*, 2015). While it remains unclear to what extent the acute stress response is suppressed in the semi-precocial storm petrel, the marked declines in baseline CORT that we observed during the course of postnatal development are consistent with the age-related decreases typical of precocial species, which usually show a robust stress response shortly after hatching (Wada, 2008).

Slow growth followed by a subsequent extension of the growth period has been widely associated with delayed fledging in birds (Bize *et al.*, 2003; Searcy *et al.*, 2004), including storm petrels (Scott, 1970), which could carry costs for both young and adults, for example, if overall energetic investment is increased and/or the start of migration is delayed. However, the observed delay in reaching near-asymptotic mass of 5 d, among high-disturbance nestlings, represents an extension of less than 8% of the typical rearing period in storm petrels, and it is typical for fledging times to vary among nests by a much larger margin both within and between years (Davis, 1957). Although strategies involving compensatory and catch-up growth have been widely shown to carry fitness costs for both parents and offspring (Dmitriew, 2011; Metcalfe and Monaghan, 2001), the observed plasticity in growth rates is likely adaptive, since the immediate survival benefit of fledging with a large body size (Krementz *et al.*, 1989; McClung *et al.*, 2004) probably has a greater impact on fitness than costs incurred later in life.

Inter-annual variation in growth trajectories proved to be of a greater magnitude than human disturbance. In 2011, which was characterized by slower growth, reproductive success was 14% lower (Watson *et al.*, 2014) and nestlings experienced accelerated telomere attrition and fledged with shorter telomeres; this is indicative of greater stress exposure and lower survival probability (Watson *et al.*, 2015), compared with 2010, collectively suggesting that environmental conditions were less favourable. Data from the nearest meteorological station (Lerwick, 33 km) revealed conditions were windier in the first half of August (coinciding with early chick rearing) in 2011 (see Watson, 2013), which could have contributed to reductions in provisioning rates and subsequently impaired chick growth. Despite the observation of inter-annual variation in growth rates and telomere dynamics, this source of environmental variability was not reflected in circulating baseline levels of CORT, which provides further evidence that baseline CORT in storm petrel nestlings is not responsive to chronic stress. Just as nestlings reared in the presence of human disturbance were able to catch up in body mass, nestlings hatched in 2011 also caught up, and in fact achieved a larger asymptotic mass, compared with those reared under what were presumably more favourable environmental conditions in 2010.

Slow postnatal growth can arise via intrinsic or extrinsic energetic constraints on the developing individual. It remains unclear whether both disturbance- and inter-annual-induced variation in growth trajectories were the result of direct stress exposure of chicks or exposure of parents to human disturbance during incubation. Although the presence of human disturbance did not alter the length of the incubation period or hatching success (Watson *et al.*, 2014), it has previously been shown that conditions during incubation can exert constraints on postnatal growth (Nilsson *et al.*, 2008). Alternatively, if adults breeding in disturbed areas perceive the presence of humans as a stressor, effects of disturbance on parents could be carried over to chick rearing and indirectly affect chick growth via effects on parental condition and provisioning behaviour (e.g. Lorentsen 1996). Yet, we did not observe the increase in baseline GCs that typically occurs under hunger (Kitaysky *et al.*, 2003; Quillfeldt *et al.*, 2007) and that might have been expected if disturbance induced a reduction in provisioning rates and/or meal sizes delivered by adults. On the other hand, storm petrel nestlings typically experience unpredictable food intake and periods of starvation and can lose up to 7 g per day (Bolton, 1995), and thus storm petrel young may not regulate CORT levels in response to energetic demands and specifically hunger in the same way as other species. Another possible mechanism, that we cannot exclude, is the possibility that high-disturbance areas were occupied by young inexperienced birds, which have been shown to provision at lower levels and with reduced synchrony, compared with experienced pairs. None of the proposed mechanisms are mutually exclusive, and they could be acting in tandem to influence postnatal growth rates in response to human disturbance.

Despite some evidence for growth-rate depression and an extended growth period, storm petrel nestlings exposed to human disturbance were able to overcome early constraints and fledge at a similar—or even higher—body mass to young that were not subject to tourism activities. The benefit of fledging with a high body mass is likely to outweigh any potential costs associated with catch-up growth or an extended rearing period. While there was no concrete evidence for differences in baseline CORT, disturbed nestlings could have experienced elevated CORT exposure if they increased their stress reactivity. Under the current level of disturbance in the study population, the effects of wildlife tourism are considered to be minimal. However, the impacts of human disturbance could be exacerbated under scenarios of elevated tourist pressure or if populations are exposed to multiple pressures (e.g. non-native predators, large-scale oceanographic anomalies). Furthermore, as it has been shown that mortality rates in disturbed areas are higher (Watson *et al.*, 2014), the surviving young and their parents are likely to represent individuals of particularly high quality. Given the increasing pressures exerted by nature-based tourism and the potential short- and long-term costs associated with sub-optimal growth, it is critical that scientists and practitioners afford more attention to the potential effects of exposure to chronic human disturbance during early life and the consequences for individual fitness and population viability. Populations of cavity-nesting procellariiform seabirds subject to higher levels of disturbance warrant closer study to determine the proximate and ultimate mechanisms underlying disturbance-induced variation in growth rates and consequences for fitness of parents and offspring.

## Funding

This work was supported by the Biotechnology and Biological Sciences Research Council (BB/F016700/1 to H.W.), the European Research Council (AdG 268926 to P.M.) and the Louise Hiom Trust (to H.W.).

## Author contributions

H.W., P.M. and M.B. designed the study. H.W. carried out fieldwork, labwork, statistical analysis and wrote the manuscript. M.B. assisted with fieldwork. B.J.H. supported labwork. All authors contributed to interpretation and writing the manuscript.

## Competing interests

The authors declare no competing interests.
